# Identifying Key Predictors of Sarcopenic Obesity in Italian Severely Obese Older Adults: Deep Learning Approach

**DOI:** 10.3390/jcm14093069

**Published:** 2025-04-29

**Authors:** Leticia Martins Cândido, Jun-Hyun Bae, Dae Young Kim, Munkh-Erdene Bayartai, Laura Abbruzzese, Paolo Fanari, Roberta De Micheli, Gabriella Tringali, Ana Lúcia Danielewicz, Alessandro Sartorio

**Affiliations:** 1 Graduate Program in Public Health, Federal University of Santa Catarina, Florianópolis 88040-900, SC, Brazil; leticia.candido96@gmail.com; 2 Institute of Sport Science, Seoul National University, Seoul 08826, Republic of Korea; baexx068@snu.ac.kr; 3 Senior Exercise Rehabilitation Laboratory, Department of Gerokinesiology, Kyungil University, Gyeongsan 38428, Republic of Korea; daeyoung@kiu.ac.kr; 4 Department of Physical and Occupational Therapy, School of Nursing, Mongolian National University of Medical Sciences, Ulaanbaatar 14210, Mongolia; munkh-erdene@mnums.edu.mn; 5 Division of Auxology, Istituto Auxologico Italiano, IRCCS, 28824 Piancavallo-Verbania, Italy; l.abbruzzese@auxologico.it; 6 Division of Pneumology, Istituto Auxologico Italiano, IRCCS, 28824 Piancavallo-Verbania, Italy; p.fanari@auxologico.it; 7 Experimental Laboratory for Auxo-Endocrinological Research, Istituto Auxologico Italiano, IRCCS, 28824 Piancavallo-Verbania, Italy; r.demicheli@auxologico.it (R.D.M.); g.tringali@auxologico.it (G.T.); sartorio@auxologico.it (A.S.); 8 Laboratory of Aging, Resources and Rheumatology, Department of Physiotherapy, Federal University of Santa Catarina, Araranguá 88906-072, SC, Brazil

**Keywords:** sarcopenic obesity, key predictors, deep learning approach

## Abstract

**Background/Objectives**: Sarcopenic obesity (SO), the coexistence of sarcopenia and obesity, poses serious health risks, such as increased mortality. Despite its clinical significance, key predictors of SO remain unclear, especially in severe obesity. This study aimed to identify independent predictors of SO in Italian older adults with obesity using a deep learning neural network. **Methods**: A cross-sectional study was conducted with hospitalized older adults diagnosed with severe obesity. SO was defined according to the 2022 ESPEN/EASO Statement Criteria, based on skeletal muscle function assessed by the five-repetition sit-to-stand test (5-SST) and body composition parameters evaluated using Dual X-ray Absorptiometry. A total of 42 independent variables were analyzed. Data normalization was performed using MinMaxScaler, and an optimal neural network architecture was selected via grid search with stratified 5-fold cross-validation. Model performance was assessed using accuracy, precision, recall, F1-score, AUC-ROC, and AUPRC metrics. **Results**: The correlation analysis revealed strong negative associations between SO and handgrip strength (HGS) (r = −0.785) and appendicular lean mass (ALM) (r = −0.745), as well as moderate correlations with 5-SST (r = 0.603), 30-second chair stand test (r = −0.474), 6-minute walking test (6m-WT) (r = 0.289), and waist circumference (WC) (r = 0.127). The deep learning model achieved an average classification accuracy of 72%, with a precision of 83% and an AUC of 0.9333. **Conclusions**: The main key predictors of SO were HGS, ALM, 5-SST, 30s-SST, 6m-WT, and WC in the early detection of this condition. The findings highlight deep learning’s potential to improve SO diagnosis, risk assessment, clinical decision-making, and prevention in severely obese older adults.

## 1. Introduction

The accelerated aging of the global population highlights the health of older adults as a central issue [1], with a particular emphasis on the risks of sarcopenia and obesity, which are prevalent conditions in this age group [2]. These conditions play a significant role in increasing morbidity and mortality [3].

Sarcopenia is characterized by the progressive and generalized loss of skeletal muscle mass and function, while obesity is the excessive accumulation of adipose tissue [4]. When these conditions coexist, the condition is known as sarcopenic obesity (SO) [5], which is considered more severe than experiencing either condition separately [6]. A systematic review and meta-analysis found that the global prevalence of SO is 11% among older adults [7]. However, in our previous study, the prevalence of SO ranged between 23.3% and 40.0% in Italian older adults with obesity, depending on the different diagnostic methods analyzed [8]. SO is associated with an increased risk of cardiovascular diseases [9], functional disability [10,11], frailty [12], and higher all-cause mortality [13].

While the literature has identified several risk factors associated with the isolated development of sarcopenia and obesity [2,14], there are still knowledge gaps regarding the predictors of SO. A review study by Pinel et al. (2024) aimed to identify the factors most frequently related to SO, revealing that insulin resistance, lack of physical activity, dyslipidemia, inflammation, and hypertension were the most commonly reported factors [15]. However, this investigation did not assess the magnitude of the effects of these factors, making it impossible to determine the statistical relevance of each. Additionally, it did not consider the new consensus for standardizing SO assessment [5].

Thus, research on SO predictors remains limited, underscoring the need for further studies to understand this condition better, implement appropriate preventive measures, and facilitate the detection and diagnosis of at-risk patients in clinical and hospital settings. Nevertheless, artificial intelligence (AI) techniques have gained prominence in predicting various diseases, including sarcopenia and SO [16,17,18], being applied to develop predictive models based on different analytical approaches.

Therefore, this study aimed to identify independent factors contributing to the diagnosis of SO in Italian older adults with severe obesity through sequential analysis using a deep learning network.

## 2. Materials and Methods

### 2.1. Study Design and Eligibility Participants

A cross-sectional study was conducted among Italian older adults of both sexes with severe obesity, who were hospitalized between April 2023 and November 2023 at the Division of Pneumological Rehabilitation and the Division of Rehabilitative Medicine, Istituto Auxologico Italiano, IRCCS, Piancavallo-Verbania, Italy.

The sample size was determined a priori based on an expected prevalence of obesity of 30% among older adults. Using the exact binomial method, a minimum of 80 participants was required to estimate this proportion with a 95% confidence interval and a total width of 0.20. The sample size calculation was performed using PASS 21 Power Analysis and Sample Size Software (2021) (NCSS, LLC, Kaysville, UT, USA). Ultimately, a total of 90 participants were recruited, which was considered sufficient to ensure adequate precision for prevalence estimates and to explore associations between the main variables under investigation.

Patients were interviewed within the first three days of hospitalization, prior to undergoing a three-week integrated multidisciplinary weight reduction program that included a calorie-restricted diet combined with physical rehabilitation, psychological counseling, and nutritional education [19]. Accordingly, all variables analyzed in the present study were collected before the commencement of the hospital-based weight reduction intervention.

The inclusion criteria were as follows: age ≥ 60 years and Body Mass Index (BMI) ≥ 35 kg/m^2^ (i.e., grade 2 and 3, according to the World Health Organization) [20]. Individuals who had prosthetics, a complete inability to walk, or any severe clinical condition that would prevent getting out of bed and/or engaging in moderate physical efforts independently were excluded.

Further details regarding the data collection methodology have been described in more detail in the previous article [8].

This study was approved by the Ethical Committee of Istituto Auxologico Italiano, IRCCS, Milan, Italy (protocol number: 2023_03_21_07; research code: 01C313; acronym: PREFISAR) and was conducted in accordance with the principles of the Declaration of Helsinki. Written informed consent was obtained from all participants prior to their inclusion in the study.

### 2.2. Dependent Variable

Sarcopenic obesity (SO) was defined according to the criteria proposed in the 2022 ESPEN/EASO consensus statement [5]. Skeletal muscle function was assessed using the five-repetition sit-to-stand test (5-SSt), while body composition was evaluated based on the concurrent presence of excess fat mass (FM%) and low muscle mass, estimated by the ratio of appendicular lean mass to body weight (ALM/W%). Both parameters were measured using Dual X-ray Absorptiometry (DXA), with a Hologic Discovery Wi device (Hologic Inc., Waltham, MA, USA) [21].

The 5-SSt involved recording the time, in seconds, taken by participants to complete five consecutive sit-to-stand movements from a chair with their arms crossed over the chest. A time greater than 17 seconds was considered indicative of altered muscle function for both genders [22]. Regarding body composition, excess FM was defined as values above 40.7% in females and 27.3% in males [23]. Low muscle mass was defined by ALM/W% values below 19.4% for females and 25.7% for males [24].

### 2.3. Independent Variables

This study considered 42 independent variables, representing a broad range of sociodemographic, lifestyle, clinical characteristics, and physical performance tests. Initially, variables were identified a priori through a comprehensive review of the literature as being relevant to SO in older adults. Additionally, these variables were available and consistently recorded in the database used for this study. Subsequently, preliminary analyses employing artificial intelligence-based methods were applied to refine the selection. This feature selection process aimed to identify variables with the highest predictive contribution and adequate statistical properties for modeling, ensuring data quality and enhancing the robustness of the final deep learning architecture. So, the following variables serve as predictors in the analysis, contributing to understanding of their associations with SO diagnostic:

- Sociodemographic characteristics included sex (female/male), age (years), level of education (elementary, middle, or high graduation), marital status (single, divorced, married, or widowed), current employment status (yes/no), and retirement status (yes/no).
- Lifestyle characteristics included alcohol consumption (never, monthly or less, 2/4 times a month, and ≥ 4 times a week); smoking (never smoked, Former smoker, and currently smoking); and regular physical activity before hospitalization (yes or no).
- Clinical and anthropometric characteristics included the sum of morbidities (total number of chronic conditions including back pain, arthritis, cancer, diabetes, hypertension, bronchitis or asthma, sleep apnea, cardiovascular disease, kidney failure, brain stroke, osteoporosis, labyrinthitis, and urinary incontinence); reported fall last year (yes/no); height (cm); weight (kg); Body Mass Index (BMI/kg/m^2^); waist circumference (WC/cm); systolic blood pressure (mmHg); diastolic blood pressure (mmHg); lean mass total DXA (LM/kg); fat mass total DXA (FM%); and appendicular lean mass total DXA (ALM/kg).
- Physical performance tests included the 6-minute walking test (6m-WT): the distance the participant can walk in six minutes, measured in meters; hand grip strength (HGS), measured in the dominant hand by a dynamometer (Lafayette Instrument, Inc., Lafayette, LA, USA); stair climb test [25]: the time taken by the participant to climb a set of stairs, measured in seconds; short physical performance battery (SPPB) [26] components: standing balance (SB), Walk 4 meters–time and speed scores (4m-WT), sit-to-stand five repetitions (5-SST), with a SPPB Total Score; physical performance test (PPT) [27] components: Write A Sentence, Simulated A Feeding, Pick A Book, Put On A Jacket, Pick Up A Coin on The Ground, 360-degree Turn, Walking 15 meters, resulting in a total score for the physical performance test; senior fitness test (SFT) [28] components: 30-second chair stand (30s-SST), 30-second arm curl, 2-minute Step Test, Chair Sit and Reach, Back Scratch, Get Up and Go Test).

All clinical, anthropometric, and physical performance tests were included in the model as continuous variables, without applying literature-based cutoffs, to retain data granularity and allow the deep learning algorithm to model complex, nonlinear associations with SO. While this limits immediate threshold-based interpretation, it aligns with the data-driven nature of the analytical approach.

### 2.4. Correlation, Variance Inflation Factor, and Tolerance Analysis

A Pearson correlation analysis was conducted to examine the linear relationships between independent variables and dependent variables in the dataset. The Pearson correlation coefficient (*r*) was calculated for each pair of variables using the Pandas “.corr()” method, which produces a correlation matrix. This matrix provided insights into the strength and direction of relationships between variables, mainly focusing on variables related to physical performance, SO diagnosis, and body composition measures. Results of the correlation analysis were reviewed to identify significant associations, which were then reported and interpreted based on standard correlation coefficient thresholds, with *r* > 0.8, indicating strong positive correlation, and *r* < −0.8, indicating strong negative correlation.

Variance inflation factor (VIF) and tolerance values were calculated to evaluate multicollinearity among the independent variables. VIF quantifies the degree of multicollinearity by measuring how much the variance of a regression coefficient is inflated due to collinearity with other predictors. Tolerance, the inverse of VIF, provides an additional metric for assessing the same. A VIF greater than 10 [29] and a tolerance value below 0.1 [30] were used as thresholds to identify multicollinearity issues [31].

### 2.5. Data Normalization and Sampling

Data were normalized using “MinMaxScaler” to avoid over-reliance on certain features during sequential deep learning neural network analysis (all variables to 0–1). Datasets were balanced via under-sampling using “RandomUnderSampler” (random state = 42) by reducing oversampling between “Non-Sarcopenia” and “Sarcopenic Obesity” (a train: test split = 80:20) [16].

### 2.6. Grid Search CV Analysis in Dataset

To optimize the deep learning of neural network architecture for the classification task, a grid search was conducted using a Keras-based model (v2.15.0) wrapped with the “KerasClassifier” from the “scikeras” library. The model architecture consisted of two dense layers with ReLU activation and an output layer with sigmoid activation for binary classification. The hyperparameters tuned through grid search included the number of neurons in the first and second dense layers (12, 16, and 20 for the first and 8, 12, and 16 for the second), dropout rates (0.0 to 0.3), learning rates (0.001 to 0.1), batch sizes (5, 10, 20, and 40), and the number of epochs (100, 200, and 300) [5,6]. Random under-sampling was applied to balance the dataset before splitting it into training and testing sets [16]. The grid search employed 3-fold cross-validation to identify the optimal combination of hyperparameters, ensuring robust model performance [32].

### 2.7. Neural Network Model and Cross-Validation Analysis

**Figure 1:**
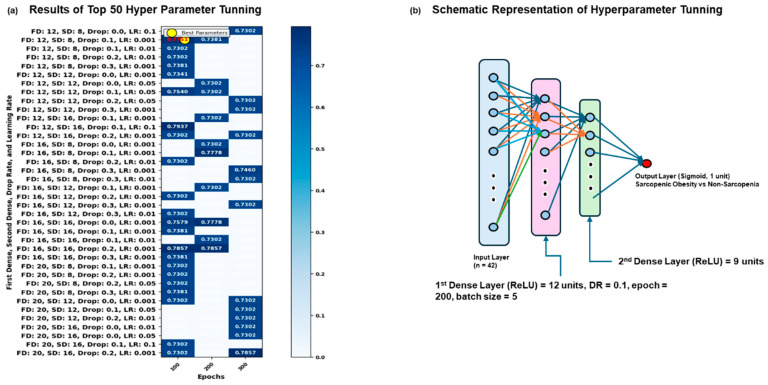
The results of grid search CV. This figure indicates the results of the grid search CV in the dataset. (**a**) These results used a deep learning for neural network model optimized through grid search cross-validation, incorporating Random Under Sampling to address class imbalance (train and test size ratio: 80:20), to identify the optimal combination of dropout rate (0.0, 0.1, 0.2, and 0.3), learning rate (0.001, 0.01, 0.05, and 0.1), batch size (5, 10, 20, and 40), and epochs (100, 200, and 300) for accurately predicting the binary target variable. (**b**) This image indicated the schematic representation of optimized hyperparameter tuning from the grid search CV. Abbreviation: FD = First Dense Layer, SD = Second Dense Layer, Drop = Drop Rate, LR = Learning Rate.

The optimal neural network architecture was determined through a grid search optimization, which evaluated multiple configurations via cross-validation (Figure 1a). The best-performing model had a first dense layer size of 12, a second dense layer size of 9, a dropout rate of 0.1, and a learning rate of 0.001. The model was trained with a batch size of 5 for 100 epochs (Figure 1b). This configuration achieved the highest performance with a mean test score of 0.7751 and a standard deviation of 0.0280, reflecting consistent results across different cross-validation folds. The moderate model size, minimal dropout, and low learning rate likely contributed to a balanced model that effectively generalizes the data.

### 2.8. Cross-Validation and Model Training in Dataset

The model was validated using 5-fold, stratified, k-fold cross-validation to maintain class balance across folds, with 80% of the data used for training and 20% for validation in each fold [16]. To avoid overfitting, the final model was trained using early stopping, with validation loss monitored and training halted if no improvement was observed over 20 consecutive epochs. The best-performing model, as determined by validation metrics, was preserved using model checkpointing [33]. The grid search, optimized by hyperparameters, was applied to the model configuration, and training was performed for up to 100 epochs with a batch size of 5. The model’s final performance was quantified through a set of evaluation metrics: accuracy, precision, recall, F1-score, AUC-ROC (area under the receiver operating characteristic curve), and AUPRC (area under the precision-recall curve). The results demonstrated that the model achieved a successful balance between complexity and generalization.

### 2.9. Performance Evaluation of Model Training and Validation in Dataset

To evaluate the model’s performance, multiple metrics were employed, including accuracy, precision, recall, F1-score, as well as the AUC-ROC, and AUPRC. Furthermore, prediction errors were examined through the calculation of mean absolute error (MAE) and mean squared error (MSE). ROC curves were generated and averaged across folds, and a confusion matrix was constructed to visualize classification performance. Additionally, the final model’s performance on precision-recall curves was analyzed to assess its ability to handle imbalanced data [34,35,36].

## 3. Results

### 3.1. The Results of Correlation, VIF, and Tolerance in Dataset

**Figure 2:**
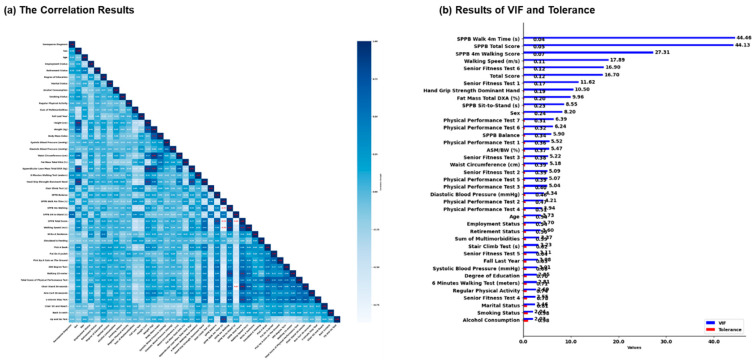
The results of correlation, variance inflation factor (VIF), and tolerance in each variable. (**a**) This figure indicated that a Pearson correlation analysis (r) was performed to assess the linear relationships between independent variables and the dependent variable, sarcopenic obesity diagnosis, which were then reported and interpreted based on standard correlation coefficient thresholds, *r* > 0.8, indicating strong positive correlation, and *r* < −0.8, indicating strong negative correlation. (**b**) This figure describes multicollinearity, which was assessed using VIF and tolerance values, with VIF quantifying the variance inflation due to collinearity among predictors. Thresholds of VIF > 10 and tolerance < 0.1 were used to identify multicollinearity issues.

The correlation analysis (Figure 2) revealed that HGS (r = −0.785) and ALM/DXA (r = −0.745) demonstrated strong negative correlations with SO diagnosis, indicating that reduced muscle strength and lean mass are highly associated with SO. In addition, the SPPB sit-to-stand test (r = 0.603) showed a moderate positive correlation, suggesting poorer physical performance in individuals with SO.

Other moderate associations were found with 30-second chair stand test (r = −0.474), 6-minute walking test (r = 0.289), and waist circumference (WC) (r = 0.127), indicating that increased waist circumference and lower physical test performance are linked to a higher likelihood of SO. Overall, the negative and positive correlation levels did not have a threshold (r > 0.8 or r < −0.8).

The VIF and tolerance analysis revealed that the SPPB Walk 4m Time (VIF = 44.46, Tolerance = 0.045), SPPB Total Score (VIF = 44.13, Tolerance = 0.045), and SPPB 4m Walking Score (VIF = 27.31, Tolerance = 0.073) exhibited particularly high multicollinearity, indicating significant overlap in explanatory power among these variables. Based on these results, the three variables with the highest multicollinearity—SPPB Walk 4m Time, SPPB Total Score, and SPPB 4m Walking Score—were removed from the model to improve its performance and interpretability, reducing multicollinearity and ensuring more reliable statistical results.

### 3.2. Results of Grid Search Analysis in Dataset

The grid search optimization identified the optimal hyperparameters for the neural network model by evaluating various configurations through cross-validation (Figure 1a). The model, with a first dense layer size of 12, second dense layer size of 9, a dropout rate of 0.1, a learning rate of 0.001, with a batch size of 5, and trained over 200 epochs (Figure 1b), achieved the highest performance with a mean test score of 0.7751 (Figure 1a). The corresponding standard deviation for this configuration was 0.0280, indicating a relatively consistent performance across cross-validation folds. These results suggest that a moderately sized network with no dropout and a lower learning rate performs best for this classification task, likely due to the balance between model complexity and generalization.

### 3.3. Results of Sequential Deep Neural Network Model Results

**Figure 3:**
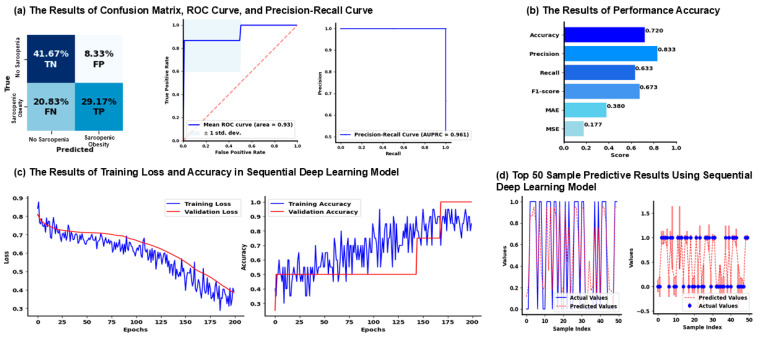
Results of the sequential deep learning model. The figure implements a neural network model trained using an under-sampled dataset to evaluate performance through metrics like accuracy, precision, recall, F1-score, ROC AUC, and AUPRC, along with visualizations such as ROC curves, performance metrics bar plots, and confusion matrix heatmaps. (**a**) The results of the confusion matrix, ROC curve, and precision-recall curve are shown. (**b**) The results of performance accuracy in the sequential deep learning model are described. (**c**) Overfitting and underfitting are assessed by comparing the loss and accuracy trends from the training and validation datasets in the sequential deep learning model. (**d**) Predicted and actual values (0 = Normal, 1 = SO) are compared, and a subset of these values is selected to display the model’s prediction accuracy for the first 50 samples using the actual data. The magnitude of the prediction error is represented by the actual value, with the error bar indicating the extent of the discrepancy. Abbreviations: Ave = average, FP = false positives, FN = false negatives, MAE = mean absolute error, MSE = mean squared error, ROC-AUC = receiver operating characteristic-area under curve, TN = true negatives, and TP = true positives.

The neural network model demonstrated strong performance in classifying SO, achieving an average accuracy of 72% (Figure 3b). The average precision was high at 0.8333, indicating that 83% of the cases predicted as SO were correct. In contrast, the average recall was lower at 0.6333, meaning the model correctly identified 63% of the actual SO cases. This balance between precision and recall resulted in an average F1-score of 0.6733, reflecting the trade-off between the two metrics. The confusion matrix (Figure 3a) further highlights the model’s ability to distinguish between sarcopenic and non-sarcopenic individuals. The true negative rate (TN) was 41.67%, while the true positive rate (TP) was 29.17%, indicating the model’s performance in both detecting SO and avoiding false positives. The average ROC-AUC of 0.9333 and AUPRC of 0.9611 emphasize the model’s ability to handle imbalanced data effectively, with a strong capacity to differentiate between classes. Overall, the model achieved a good balance of precision and recall, with consistent performance across cross-validation.

### 3.4. Results of Validation and Training Results of Sequential Deep Neural Network Model

The training and validation curves (Figure 3c) illustrate the model’s performance over 200 epochs. In the left plot, the training and validation loss both decrease steadily over time, indicating that the model is learning effectively. The validation loss closely follows the training loss, suggesting that the model generalizes well without overfitting. By the end of training, the loss reaches a relatively low value, reflecting improved performance in minimizing prediction errors. In the right plot, the training accuracy increases over time with fluctuations, reaching approximately 90% by the end of the 200 epochs. The validation accuracy, however, remains relatively steady after an initial improvement, with a final accuracy near 50%. This divergence between training and validation accuracy indicates that while the model fits well to the training data, there is a degree of overfitting, as reflected by the limited improvement in validation accuracy.

Figure 3d shows the top 50 sample predictive results using the sequential deep learning model for SO classification. The left plot compares the actual values (solid blue line) with the predicted values (dashed red line) across sample indices. The right plot further highlights this with actual values represented by blue dots and predicted values by a red dashed line, including error bars. The presence of substantial error margins in certain cases suggests that the model struggles with prediction consistency. These results indicate the model performs well on some samples in the dataset.

## 4. Discussion

The study identified key predictors for SO through the sequential deep learning network analysis. The deep neural network model, optimized through grid search, achieved strong performance, with a precision of 83% and an AUC of 0.9333. These findings identify dominant hand grip strength (HGS), total appendicular lean mass (ALM), the SPPB sit-to-stand test (5-SST), the 30-second chair stand test (30s-SSt), the 6-minute walking test (6m-WT), and waist circumference (WC) as key predictors of SO.

Both WC and ALM are important variables in the assessment of SO, as both are related to the imbalance between muscle mass and body fat, which characterizes this condition [4,5]. Evidence indicates that the accumulation of total, intramuscular, and visceral fat is associated with an increase in the secretion of pro-inflammatory adipokines and chemokines, in addition to being linked to reduced muscle function and a higher risk of mortality and comorbidities [37,38,39]. The excess of adipose tissue—especially the fatty infiltration in skeletal muscle (IMAT)—appears to impair muscle quality, contraction speed, and the specific force of muscle fibers, ultimately compromising physical performance [40,41,42,43], characteristic of the diagnosis of SO. Additionally, adipose tissue-derived inflammatory markers, such as monocyte chemoattractant protein-1 (MCP-1) [44], C-reactive protein [45], leptin, and perilipin 2 (Plin2) [46], have been associated with SO and may play a role in its pathophysiology [47]. Therefore, these findings reinforce the importance of muscle mass and function, as well as adipose tissue composition, in their systemic impact on the development of SO [47].

The present study revealed that poorer performance in the 6m-WT, HGS, and both sit-to-stand tests (5-SST and 30s-SST) are predictors of the occurrence of SO in severely obese Italian older adults. Chang et al. (2015) demonstrated that Taiwanese individuals aged 65 years or older diagnosed with isolated sarcopenia or SO presented worse physical performance (gait speed tests, HGS, and timed up and go test) compared to those without these conditions [48]. The study by Kong et al. (2020) with 2,303 community-dwelling residents aged 70 to 84 years showed lower scores on the SPPB tests in residents with SO than in those with isolated sarcopenia or obesity [49]. When these abilities are compromised, older adults tend to become more sedentary, which, in turn, accelerates muscle loss and fat gain [4], perpetuating a vicious cycle of physical inactivity [50,51,52,53]. SO is a modifiable condition; therefore, regular physical activity is crucial in preventing or delaying its development. Age-related muscle mass loss is associated with a reduction in protein production, which decreases by almost 6% per decade after middle age [50], partly due to the lack of anabolic stimuli, such as exercise. However, aerobic, resistance, or a combination of these exercise modalities can counteract this reduction by stimulating muscle synthesis despite natural body changes [51].

Additionally, exercise activates satellite cells, which are essential for muscle regeneration, promoting recovery after minor injuries [52]. Concurrently, weight gain is associated with an increase in adipocytes, a process that can trigger the infiltration of immune cells and the release of pro-inflammatory mediators in adipose tissue, such as interleukin-6, C-reactive protein, and tumor necrosis factor. This inflammatory profile contributes to a state of chronic low-grade inflammation, which negatively affects insulin sensitivity [53]. Insulin resistance hinders protein anabolism, favoring muscle fiber atrophy and promoting muscle catabolism [53].

Regular physical activity also modulates systemic inflammatory markers, whose elevation is associated with reduced strength, mobility, and muscle function. Muscle contractions induced by exercise trigger the production of nitric oxide (NO), which, in turn, enhances insulin sensitivity, supporting muscle protein synthesis and adaptation. The interaction between NO and insulin during exercise may synergistically affect muscle metabolism, helping maintain muscle mass and attenuating the effects of sarcopenia. Thus, regular exercise stands out as an effective strategy to mitigate the effects of sarcopenia and preserve muscle functionality throughout aging [52].

The American College of Sports Medicine [54] preconizes that strength training programs be performed on no fewer than two non-consecutive days per week, with sets comprising 8–12 repetitions for healthy adults and 10–15 repetitions for older and frail individuals. The meta-analysis by Eglseer et al. (2023) demonstrated that resistance training (with both slow and fast velocity exercises) performed two to three times per week, for 20 to 60 minutes, is effective in reducing body fat and increasing muscle mass, strength, and gait speed [55]. As stated in the systematic review and meta-analysis of randomized controlled trials conducted by Yoshimura et al. (2017), resistance training (e.g., walking, cycling, and swimming) should predominantly consist of one or two sets, with 8–12 repetitions at approximately 65% of the maximum load (one repetition maximum) [56,57]. The goal is to gradually progress to two or three sets at 75% of one repetition maximum over time. It is noteworthy that aerobic training should be combined with increased protein intake to reduce absolute body fat mass [consuming 1.0–1.2 g/kg of body weight, with even higher intakes (1.2–1.5 g/kg of body weight) suggested for individuals with chronic diseases] [56,57].

Furthermore, the systematic review by Bai et al. (2022) evidenced that a combination of resistance, strength, and aerobic exercise was more effective than aerobic exercise alone in improving seated position and stretching, knee flexion, elbow flexion, shoulder flexion and stretching, strength, body fat, functional reach test, 30s-SSt, and 6m-WT [58]. Therefore, the integration of multiple components into exercise routines improves overall physical fitness in older adults, playing a key role in preventing the onset of SO.

Therefore, the results from the deep learning model demonstrated high precision and recall in predicting SO based on the presence of some variables, highlighting its potential to improve the prediction of this condition through deep learning procedures. The use of AI in this research allows the identification of complex and non-linear patterns between variables, which are often inaccessible to traditional methods. While conventional models assume linear relationships and require the prior definition of interactions between variables, AI automatically learns to capture complex interactions and hidden patterns in the data, increasing predictive power (evidenced by the prediction of SO in the older adults in this study). Therefore, the future of AI in healthcare promises to offer more accurate and personalized predictions, complementing traditional approaches, and bringing a deeper understanding of the phenomena that encompass SO. However, there is still a need for the development of a more detailed and accurate SO prediction model, applicable to diverse population groups, as this study specifically focused on older Italian individuals with severe obesity.

### Limitations

The main limitation of this study is the sample consisting exclusively of Italian older adults with severe obesity, hospitalized, and undergoing a multidisciplinary body weight reduction program, which compromises external validity. The specific conditions, such as hospitalization and extreme obesity, may influence the functional outcomes and observed associations, making them unrepresentative of older adults with different levels of obesity or in different community settings. The variety of diagnostic approaches has posed a challenge for both the accurate diagnosis and effective treatment of SO, in addition to hindering comparability between studies already conducted. However, the present research utilized the ESPEN/EASO 2022 criteria, which represents the first consensus for standardizing the evaluation of SO.

## 5. Conclusions

Poor performances on the 6m-WT, HGS, and sit-to-stand tests (5-SST and 30s-SST) were associated with a higher likelihood of being diagnosed with SO. Additionally, ALM and WC were confirmed as significant predictors of SO in Italian older adults with severe obesity, highlighting the significance of including these parameters in the evaluation of SO.

## Data Availability

The data and findings presented in this study are contained within the article. For additional information or inquiries, please contact the corresponding author.

## Abbreviations

SO	Sarcopenic Obesity
IRCCS	Istituto di Ricovero e Cura a Carattere Scientifico
ESPEN	European Society for Clinical Nutrition and Metabolism
EASO	European Association for the Study of Obesity
5-SST	Five-Repetition Sit-To-Stand Test
HGS	Handgrip Strength
ALM	Appendicular Lean Mass
6m-WT	6-Minute Walking Test
30s-SST	30-Second Chair Stand Test
WC	Waist Circumference
BMI	Body Mass Index
FM	Fat Mass
ALM/W	Appendicular Lean Mass Adjusted To Body Weight
DXA	Dual X-ray Absorptiometry
SPPB	Short Physical Performance Battery
VIF	Variance Inflation Factor
AUC-ROC	Area Under The ROC Curve
AUPRC	Precision-Recall Curve
NO	Nitric Oxide
